# A case of immune thrombocytopenia complicated by catastrophic antiphospholipid antibody syndrome and subsequent intramuscular haemorrhage

**DOI:** 10.1186/s13256-026-06291-6

**Published:** 2026-07-07

**Authors:** Priyanta Banerjee, David Allsup, Elizabeth Uglow, Corinna Hauff, Bryan Jason Atienza, Jireh Ann Batac

**Affiliations:** 1 Hull University Teaching Hospitals NHS Trust, Hull, UK; 2 Centre for Biomedicine, Hull York Medical School, Hull, UK

## Abstract

**Background:**

Immune thrombocytopenia (ITP) and antiphospholipid syndrome (APS) are autoimmune disorders that may coexist, creating a challenging clinical scenario characterised by bleeding risk alongside paradoxically increased thrombotic risk. Catastrophic antiphospholipid syndrome (CAPS) is a rare but life-threatening manifestation of APS. The occurrence of CAPS in a patient with ITP receiving thrombopoietin receptor agonist therapy is uncommon and highlights important diagnostic and therapeutic challenges, particularly in the presence of concurrent bleeding manifestations.

**Case presentation:**

A 51-year-old White British woman presented with new-onset immune thrombocytopenia that initially responded to corticosteroid therapy but subsequently relapsed, requiring treatment with avatrombopag. She later developed an unprovoked pulmonary embolism and, months afterward, rapidly progressive multiorgan thrombosis involving the kidneys, spleen, adrenal glands, retina, and brain following a urinary tract infection. Laboratory evaluation demonstrated a positive lupus anticoagulant and medium-titre anti-β2-glycoprotein I antibodies, supporting a diagnosis of probable catastrophic antiphospholipid syndrome. Avatrombopag was discontinued, and treatment with therapeutic anticoagulation, high-dose corticosteroids was initiated, resulting in clinical improvement. Her subsequent course was complicated by a large intramuscular thigh haematoma during anticoagulation and adrenal insufficiency secondary to bilateral adrenal involvement. Following further management, including rituximab therapy, the immune thrombocytopenia entered complete remission, and she remains on long-term anticoagulation and endocrine follow-up.

**Conclusions:**

This case highlights the complex interplay between immune thrombocytopenia, antiphospholipid antibody positivity and thrombopoietin receptor agonist therapy. Clinicians should maintain a high index of suspicion for APS in patients with ITP who develop thrombotic events. Early recognition and treatment of CAPS are critical, even in the presence of significant thrombocytopenia, and careful balancing of thrombotic and bleeding risks is essential for optimal patient outcomes.

## Introduction

Immune thrombocytopenia (ITP) is an acquired autoimmune disorder characterised by isolated thrombocytopenia resulting from increased platelet destruction and impaired platelet production and remains a diagnosis of exclusion [1]. Although bleeding is the principal clinical concern, accumulating evidence demonstrates a paradoxical increase in thrombotic risk, even in the absence of traditional cardiovascular risk factors [2, 3]. Approximately 10–20% of patients with ITP have detectable antiphospholipid antibodies (aPL), and several studies suggest that aPL-positive ITP may represent a distinct prothrombotic phenotype that can precede the development of clinically overt antiphospholipid syndrome (APS) [4, 5]. Notably, thrombocytopenia in APS does not confer protection against thrombosis and may instead identify a subgroup with heightened thrombotic risk, reflecting ongoing immune-mediated platelet activation and consumption within a prothrombotic milieu [6].

Thrombopoietin receptor agonists (TPO-RAs), including avatrombopag, are widely used in ITP [3, 7]. Whilst effective in increasing platelet counts and reducing bleeding episodes, emergent data indicate a potential increase in thrombotic risk, particularly in patients with underlying prothrombotic autoimmune conditions, such as APS [7]. In such patients, therapeutic strategies aimed at correcting thrombocytopenia must be balanced against the risk of precipitating or amplifying thrombotic events.

Catastrophic APS (CAPS) is a rare but life-threatening variant of APS characterised by rapidly progressive thrombosis affecting three or more organ systems within a short period. Infection, surgery, and systemic immune activation are recognised precipitating factors. At the same time, the requirement for sustained therapeutic anticoagulation in CAPS creates a substantial bleeding risk, particularly in patients with concomitant thrombocytopenia [8, 9].

The case presented herein illustrates the complex interplay between ITP, aPL positivity, infection-triggered immune activation, and TPO-RA therapy, culminating in the development of CAPS, followed by a major haemorrhagic complication during anticoagulation. It highlights the profound therapeutic tension between thrombosis and bleeding in overlapping autoimmune haematological disorders.

## Case presentation

A 51-year-old white British female with a background of hypothyroidism, who was previously well and independent, presented to the haematology service in December 2024 with new-onset widespread petechiae and bruising. Symptoms developed rapidly 4 weeks after receiving the seasonal influenza vaccination. Initial blood tests demonstrated isolated severe thrombocytopenia, and other causes of thrombocytopenia were considered and evaluated. An autoimmune panel was tested and found to be negative. After exclusion of other diagnoses, the patient was diagnosed with immune thrombocytopenia (ITP). She had a haematological response to prednisolone 70 mg daily, with normalisation of the platelet count. Antiphospholipid antibody testing was not performed at that stage, in line with guidelines, which do not recommend routine screening unless there are clinical features suggestive of APS or thrombosis. Steroids were tapered over 4 weeks after 2 weeks of high-dose steroids and discontinued on 17 January 2025.

In March 2025, the patient re-presented with a symptomatic relapse of ITP, characterised by extensive bruising, malaise, mucocutaneous bleeding, and a platelet count of 1 × 10⁹/L. Given the severity of thrombocytopenia and steroid-related adverse effects, intravenous immunoglobulin (IVIg) was considered but not started, as no further bleeding manifestations were present except for an episode of epistaxis, which stopped spontaneously. Therefore, tranexamic acid was not also considered.

Avatrombopag was commenced and titrated between 20 and 40 mg on alternating days according to platelet response. Platelet counts improved, allowing steroid tapering, and she remained on avatrombopag for ongoing ITP control. A target platelet count of ≥ 50 × 10⁹/L was maintained, whilst she was treated with apixaban, and avatrombopag was administered at a dose of 20 mg once daily.

**Fig. 1:**
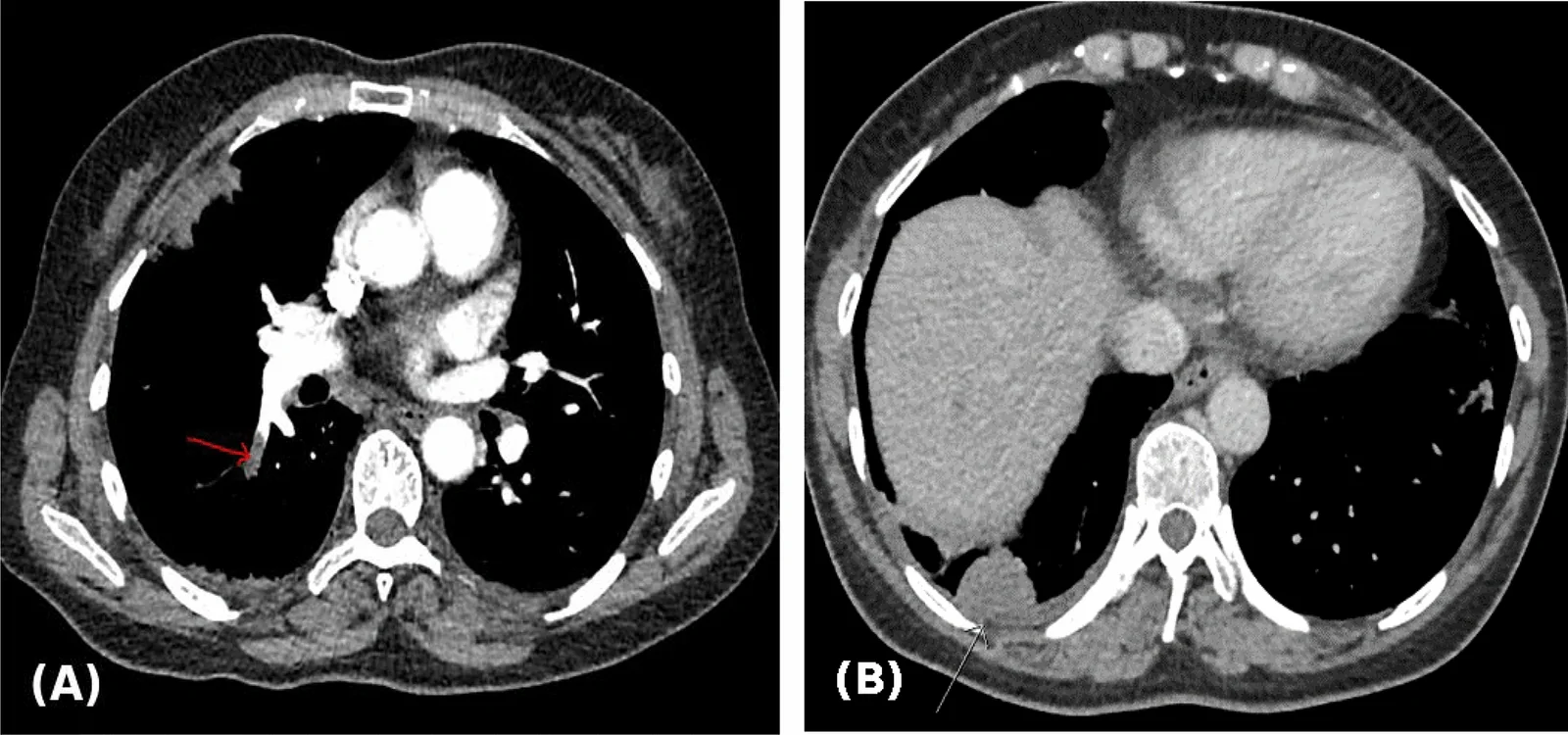
CT pulmonary angiography images showing: **A** filling defects within segmental pulmonary artery branches consistent with pulmonary embolism and **B** wedge-shaped opacity in the posterior right lower lobe consistent with pulmonary infarction

In April 2025, the patient developed acute-onset pleuritic chest pain and shortness of breath. CT pulmonary angiography demonstrated a right segmental pulmonary embolism with associated pulmonary infarcts (Fig. 1).

At the time of diagnosis, the platelet count was 50 × 10⁹/L. The patient was commenced on apixaban 5 mg twice daily for long-term anticoagulation, whilst avatrombopag was continued with a platelet target above 50 × 10⁹/L to permit safe therapeutic anticoagulation.

**Fig. 2:**
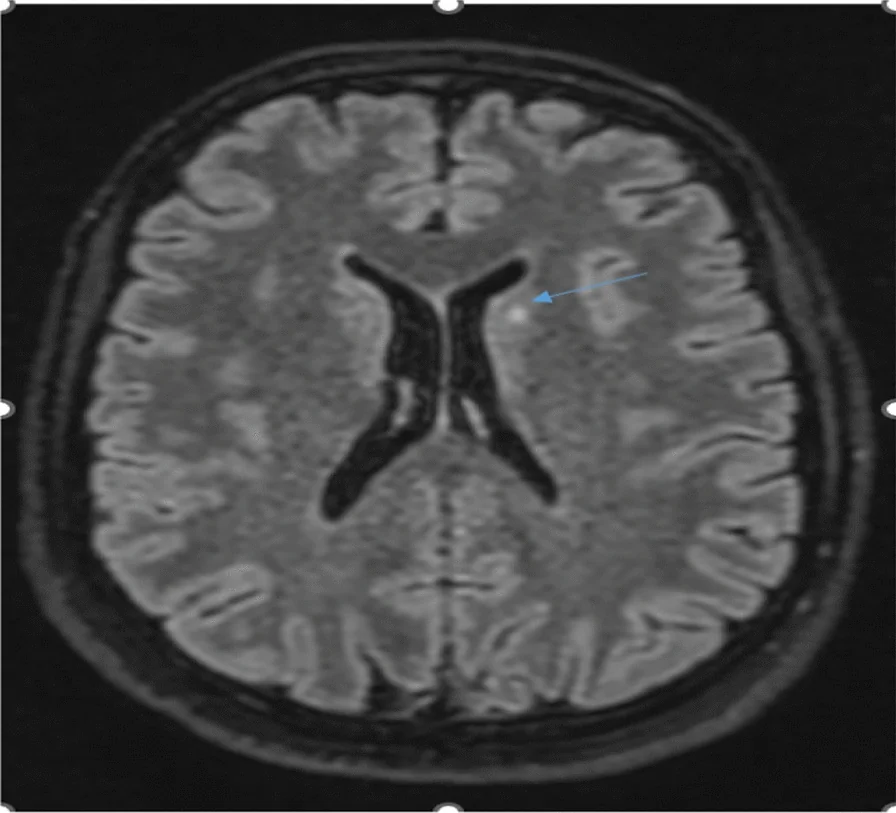
MRI brain (axial T2 FLAIR and DWI sequences) demonstrating a small hyperintense lesion in the head of the left caudate nucleus, consistent with acute infarction

The patient remained clinically stable until mid-September 2025, when she re-presented a few days after treatment for a urinary tract infection with malaise, right-sided abdominal and flank pain, a new mottled rash over her trunk, worsening headache (Fig. 2), and visual disturbance. On admission, the platelet count was 60 × 10⁹/L. Examination revealed bilateral subconjunctival haemorrhages and a livedo reticularis-type rash involving the trunk, abdomen, and thighs. She was alert, afebrile, and haemodynamically stable, with no focal neurological deficits. The abdomen was soft and non-tender.

**Fig. 3:**
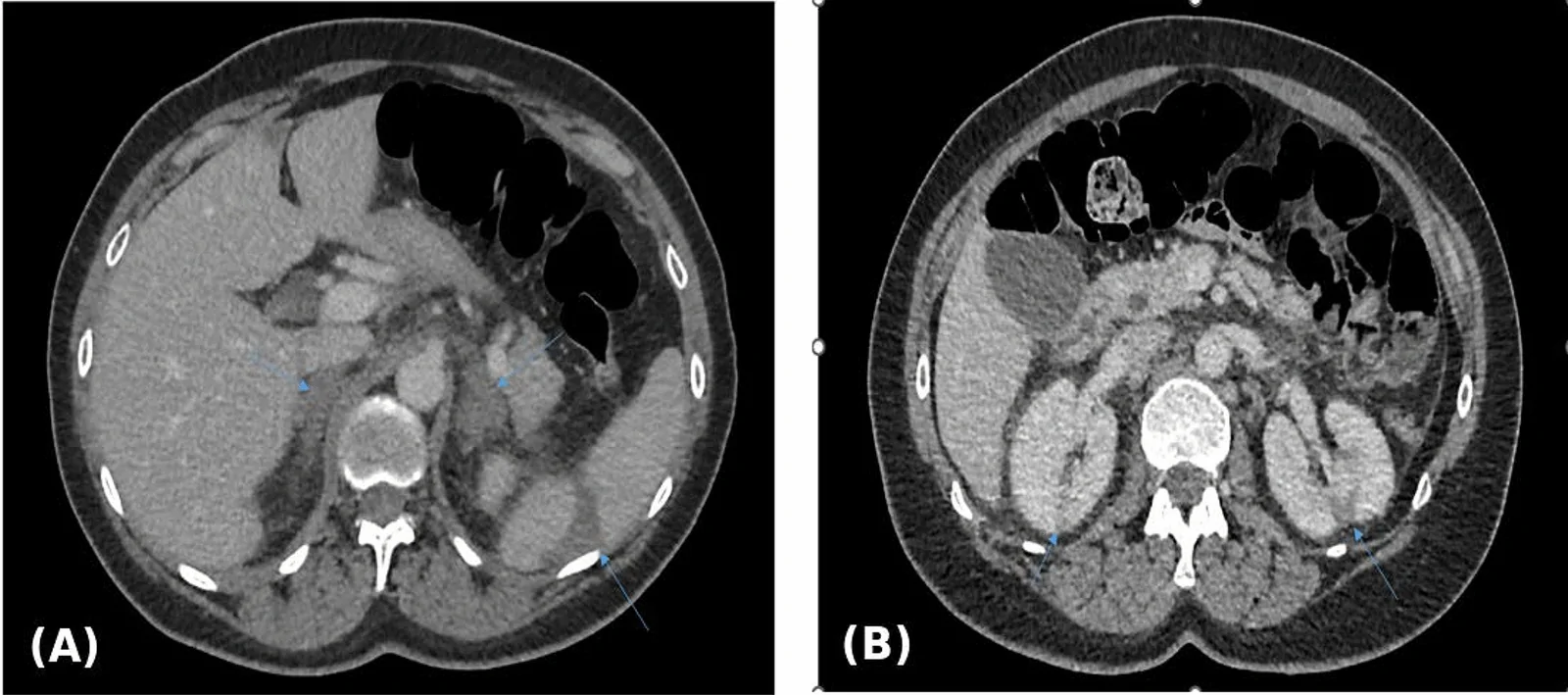
Contrast-enhanced CT abdomen and pelvis (portal venous phase) demonstrating: **A** Wedge-shaped area of low attenuation in the spleen abutting the capsule, consistent with splenic infarction and **B** focal hypoattenuation in the posterior cortex of the left kidney, consistent with renal infarction. A smaller infarct is also present in the right kidney

CT imaging of the thorax, abdomen, and pelvis demonstrated new splenic and renal infarcts, along with bilateral adrenal gland enlargement and loss of definition, raising suspicion of evolving adrenal infarction. No intra-abdominal bleeding or malignancy was identified (Fig. 3).

**Fig. 4:**
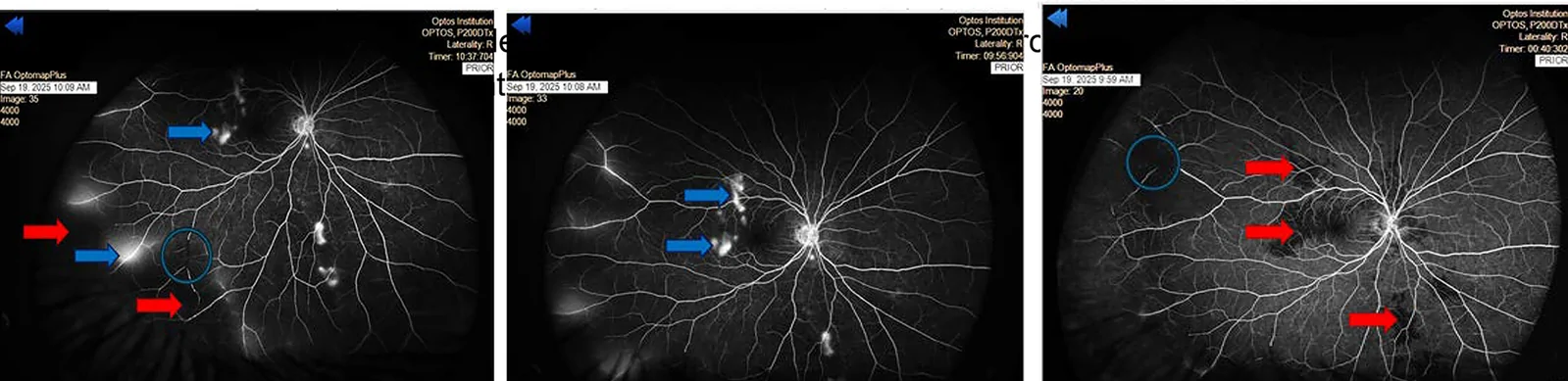
Fundus fluorescein angiography demonstrating features consistent with Purtscher-like retinopathy and microvascular occlusion: **A** early-phase image showing areas of capillary non-perfusion and patchy retinal pallor; **B** mid-phase image demonstrating persistent areas of hypoperfusion with surrounding retinal whitening; and **C** late-phase image demonstrating choroidal ischaemia and areas of leakage

During admission, progressive visual symptoms developed, including blurred vision and painful eye movements. Neuro-ophthalmological assessment identified patchy retinal pallor. Fundus fluorescein angiography confirmed Purtscher-like retinopathy with areas of choroidal ischaemia and late leakage, consistent with microvascular occlusion (Fig. 4).

Laboratory investigations revealed a platelet count of 43 × 10⁹/L, haemoglobin 116 g/L, and acute kidney injury. The blood film showed no schistocytes, and lactate dehydrogenase levels were not elevated, making thrombotic microangiopathy less likely. Antiphospholipid antibody testing demonstrated a positive anti-β2 glycoprotein I IgG antibody level of 56 (reference range 0–19.9), the presence of a moderate lupus anticoagulant, and an anticardiolipin antibody level of 19 (reference range 0–19.9). Acknowledging the possibility of false positivity due to apixaban therapy, the sample was processed using a DOAC–StopTM (Haematex Research, Hornsby, Australia) reagent to remove apixaban interference. This antiphospholipid antibody profile was considered medium titre in our laboratory. PF4-related disorders were excluded with negative anti-PF4 antibodies (rapid chemiluminescence assay and ELISA). Testing for paroxysmal nocturnal haemoglobinuria was negative. Thrombotic thrombocytopenic purpura was considered but excluded based on the absence of microangiopathic haemolytic anaemia.

The combination of prior unprovoked pulmonary embolism and new renal, splenic, adrenal, cerebral, and retinal infarctions was highly suggestive of catastrophic antiphospholipid syndrome (CAPS). The patient fulfilled criteria for probable CAPS, including involvement of three or more organ systems, rapid progression, and laboratory evidence of antiphospholipid antibodies. The recent urinary tract infection was considered a likely precipitant.

Apixaban was discontinued and therapeutic enoxaparin 80 mg twice daily was commenced despite thrombocytopenia, given the overwhelming thrombotic burden.

Aspirin 75 mg daily was added following multidisciplinary discussion due to the significant arterial involvement. Avatrombopag was discontinued. Prednisolone 1 mg/kg daily (70 mg) and intravenous immunoglobulin (2 g/kg over 2 days) were administered. Plasma exchange was considered in the event of further progression but was ultimately not required.

The patient’s symptoms gradually improved, with resolution of abdominal pain and headache. Retinal imaging demonstrated partial reperfusion. Platelet counts rose and later normalised above 400 × 10⁹/L.

The patient was discharged on enoxaparin 80 mg twice daily, based on body weight and creatinine clearance, aspirin 75 mg daily, and prednisolone 70 mg daily, with plans to transition to warfarin (target INR 2–3).

**Fig. 5:**
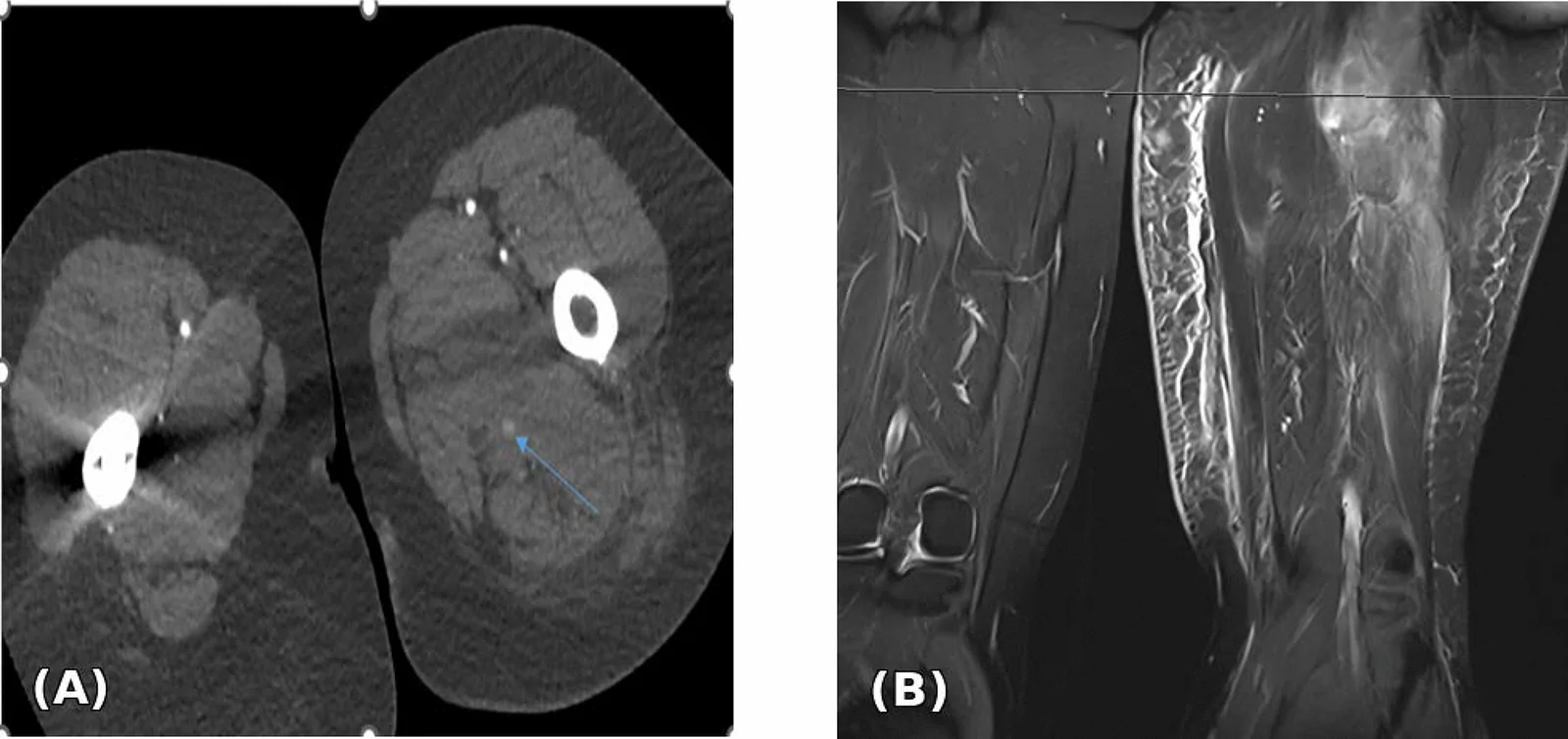
Imaging of the lower limbs demonstrating a haemorrhagic complication: **A** CT angiogram of the lower limbs showing a small focus of contrast blush in the arterial phase (arrow), consistent with active contrast extravasation from a branch of the profunda femoris artery and **B** MRI of the thighs (axial and coronal STIR sequences) demonstrating an extensive haematoma within the posterior compartment of the left thigh, with associated soft tissue swelling and subcutaneous oedema

There was a subsequent re-admission with a left-sided intramuscular thigh haematoma secondary to bleeding from a branch of the profunda femoris artery, further complicating anticoagulation management (Fig. 5). Initially, haemodynamic support was provided with intravenous fluids and the transfusion of one unit of packed red blood cells. Arterial embolisation was considered, and the case was discussed with the vascular surgery team. However, following specialist review, embolisation was deemed unsuitable in this patient. The femoral vessels were noted to be very small in calibre relative to the size of the endovascular equipment, raising concern that arterial access and catheter manipulation could precipitate further vascular injury or exacerbate bleeding. Given this unfavourable risk–benefit profile, the vascular team advised against embolisation.

In view of these technical limitations and the potential for procedure-related harm, a decision was made to pursue conservative management, with close clinical and radiological monitoring, which was felt to be the safest and most appropriate approach in this context.

Warfarin was withheld, and the patient was anticoagulated with low molecular weight heparin. Prothrombin levels were checked to exclude hypoprothrombinaemia, which can be seen in antiphospholipid syndrome, but the levels were normal. During this period, she also developed primary adrenal insufficiency secondary to bilateral adrenal haemorrhage related to CAPS. She remained under regular haematology and endocrinology follow-up with close platelet and symptom monitoring.

One month later, the patient developed recurrent petechiae and was found to have a platelet count of 1 × 10⁹/L, consistent with ITP relapse. Prednisolone was recommenced with a tapering schedule, hydrocortisone maintenance therapy was temporarily withheld, and rituximab therapy was started. Given the competing risks of bleeding and thrombosis, the anticoagulation strategy was individualised: enoxaparin was restarted at a prophylactic dose and gradually increased to a therapeutic dose of 80 mg subcutaneously twice daily, with close monitoring of bleeding symptoms and platelet count.

The patient’s anticoagulation was later transitioned back to warfarin with heparin bridging and close monitoring due to her previous bleeding complications.

At present, the patient is anticoagulated with warfarin without further bleeding. The immune thrombocytopenia is in complete remission following rituximab therapy. She remains under regular haematology follow-up every 2–3 weeks for platelet count monitoring and assessment of medication side effects. Prednisolone treatment has been discontinued, and she is currently maintained on low-dose hydrocortisone for adrenal insufficiency.

## Discussion

The present case illustrates a rare but clinically important intersection between immune thrombocytopenia (ITP), antiphospholipid antibody positivity, and thrombopoietin receptor agonist (TPO-RA) therapy, culminating in catastrophic antiphospholipid syndrome (CAPS) with subsequent haemorrhagic complications during anticoagulation. This sequence of events highlights the complex and often paradoxical relationship between thrombocytopenia, immune dysregulation, and thrombosis, and underscores the therapeutic tension that arises when prothrombotic and haemorrhagic risks coexist in the same patient.

Although ITP has traditionally been regarded as a disorder characterised primarily by bleeding due to immune-mediated platelet destruction, a growing body of evidence suggests that patients with ITP have an increased risk of thrombosis compared with the general population [2, 3, 10]. Several mechanisms have been proposed to explain this paradoxical prothrombotic tendency. These include increased circulating platelet-derived microparticles, immune-mediated endothelial activation, elevated levels of procoagulant cytokines, and the presence of young, hyperreactive platelets released in response to accelerated platelet turnover [3, 11]. In addition, treatments for ITP, particularly corticosteroids, splenectomy, and thrombopoietin receptor agonists, may further augment thrombotic risk through effects on platelet production, endothelial activation, and coagulation pathways [3, 7].

Antiphospholipid antibodies are detected in approximately 10–20% of individuals with ITP, and their presence may identify a distinct subgroup of patients with heightened thrombotic susceptibility [4, 5]. This may influence both aetiological understanding and therapeutic decisions. This case could guide as a basis for encouraging aPL testing in patients with ITP. In some cases, ITP may represent an early or incomplete manifestation of APS, preceding overt thrombotic events by months or years. The initial presentation of isolated thrombocytopenia in APS is well-recognised and may complicate diagnostic interpretation, particularly when thrombosis has not yet occurred [5, 12]. In the present case, the patient initially presented with isolated thrombocytopenia and was treated according to standard ITP management pathways. However, the subsequent development of an unprovoked pulmonary embolism strongly suggests that pathogenic antiphospholipid antibody activity was already present before the later catastrophic presentation. This case underpins the need for early consideration of antiphospholipid syndrome in patients presenting with venous thromboembolism and thrombocytopenia, particularly given current guidance regarding the use of DOACs in antiphospholipid antibody-positive patients.

The subsequent evolution to CAPS is particularly notable. CAPS is a rare but highly lethal variant of APS characterised by rapidly progressive small- and medium-vessel thrombosis affecting multiple organ systems within a short time frame. The pathogenesis of CAPS is thought to involve a “thrombo-inflammatory storm” in which antiphospholipid antibodies, complement activation, endothelial injury, and systemic inflammatory triggers interact to produce widespread microvascular thrombosis. Infection is among the most frequently reported precipitating factors, accounting for approximately one-third of CAPS cases. In this patient, the temporal association between a recent urinary tract infection and the rapid onset of multiorgan thrombosis suggests that infection-driven immune activation may have served as the precipitating trigger for CAPS [8, 9].

Another important consideration in this case is the potential role of thrombopoietin receptor agonist therapy. TPO-RAs such as avatrombopag have transformed the management of refractory ITP by stimulating megakaryopoiesis and increasing platelet production. However, their use has been associated with an increased incidence of thromboembolic events [3, 7]. The underlying mechanisms remain incompletely understood but may include increased production of large, young platelets with enhanced procoagulant activity, increased thrombin generation, and elevated levels of circulating platelet microparticles. In patients with an underlying prothrombotic autoimmune condition, such as APS, the prothrombotic effects of TPO-RA therapy may theoretically amplify an already activated coagulation environment. Although causality cannot be definitively established in a single case, the temporal relationship between avatrombopag exposure, the occurrence of pulmonary embolism, and subsequent widespread thrombosis raises the possibility that TPO-RA therapy may have contributed to thrombotic amplification in this patient.

Management of CAPS in the context of thrombocytopenia presents a particularly challenging clinical dilemma. Despite severe thrombocytopenia, anticoagulation remains a cornerstone of therapy, because uncontrolled thrombosis represents the principal driver of organ failure and mortality in CAPS. Current therapeutic strategies typically involve a combination of full-dose anticoagulation, high-dose corticosteroids, and immunomodulatory therapy, such as intravenous immunoglobulin or plasma exchange. In the present case, prompt recognition of rapidly progressive multiorgan thrombosis allowed early initiation of therapeutic anticoagulation and immunosuppression, resulting in clinical stabilisation and partial organ recovery.

From a diagnostic perspective, this case reinforces the importance of reconsidering the underlying aetiology when patients with presumed primary ITP develop thrombotic complications. The occurrence of unexplained thrombosis, livedo reticularis, or other APS-associated features should prompt evaluation for antiphospholipid antibodies. Early identification of aPL positivity may influence both therapeutic decisions and long-term risk stratification. Syk inhibitors, particularly fostamatinib, are approved for refractory ITP and reduce antibody-mediated platelet destruction without increasing thrombotic risk. They may represent a safer alternative for patients at high thrombotic risk compared with TPO-RAs.

The subsequent development of a large intramuscular haematoma during anticoagulation illustrates the competing haemorrhagic risks that often accompany treatment in this setting. Patients with overlapping autoimmune haematological disorders may experience rapid fluctuations in platelet counts and coagulation status, as well as vessel fragility, making anticoagulation management particularly complex. In our patient, warfarin was discontinued following the development of an expanding thigh haematoma, and anticoagulation was temporarily withheld with close haemodynamic and laboratory monitoring. Once haemoglobin stabilised and there was no further evidence of active bleeding, anticoagulation was cautiously reintroduced using low molecular weight heparin with gradual dose escalation under close clinical surveillance, reflecting the exceptionally high ongoing thrombotic risk associated with catastrophic APS. Importantly, the bilateral adrenal haemorrhage observed in this case was felt to represent adrenal vein or microvascular thrombosis with secondary haemorrhagic infarction, a recognised manifestation of CAPS rather than a primary bleeding phenomenon alone. Bilateral adrenal involvement in APS and CAPS is well-described as resulting from thrombotic vascular occlusion leading to venous congestion and secondary haemorrhage, and anticoagulation remains a cornerstone of therapy to prevent ongoing thrombosis and further organ damage. The subsequent development of adrenal insufficiency further reflects the severe systemic vascular injury characteristic of CAPS and highlights the importance of careful multidisciplinary management in balancing catastrophic thrombotic risk against major haemorrhagic complications.

Taken together, this case highlights the complex interplay between immune-mediated thrombocytopenia, antiphospholipid antibody-driven thrombosis, inflammatory triggers, such as infection, and the prothrombotic potential of certain ITP therapies. Recognition of this intersection is essential for clinicians managing patients with refractory thrombocytopenia who develop unexpected thrombotic events.

## Conclusion

This case demonstrates the potentially hazardous overlap between immune thrombocytopenia and antiphospholipid antibody-mediated thrombosis, culminating in catastrophic antiphospholipid syndrome with subsequent haemorrhagic complications during anticoagulation. The progression from isolated thrombocytopenia to unprovoked venous thrombosis and ultimately to multiorgan arterial and microvascular thrombosis illustrates how unrecognised antiphospholipid antibody positivity, inflammatory triggers such as infection, and immune-directed therapies may converge to produce severe thrombotic disease.

Early recognition of rapidly progressive multiorgan thrombosis remains critical for improving outcomes in catastrophic APS. Prompt initiation of therapeutic anticoagulation, immunosuppressive therapy, and multidisciplinary management can be lifesaving, even in the presence of significant thrombocytopenia.

More broadly, this case supports consideration of antiphospholipid antibody testing in patients with ITP who develop unexplained thrombosis or atypical clinical features. Greater awareness of the potential overlap between ITP and APS may facilitate earlier diagnosis, guide therapeutic decision-making, and ultimately reduce the risk of life-threatening thrombotic complications.

## Data Availability

All data used and analysed during this study are included in this case report.

## Declarations

Ethics approval and consent to participate

Written consent was obtained from the patient. Ethics approval was not applicable. All identifiable patient images were excluded.

## References

[1] Neunert, C., Terrell, D.R., Arnold, D.M., Buchanan, G., Cines, D.B., Cooper, N., Cuker, A., Despotovic, J.M., George, J.N., Grace, R.F., Kühne, T., Kuter, D.J., Lim, W., McCrae, K.R., Pruitt, B., Shimanek, H. and Vesely, S.K., American Society of Hematology 2019 guidelines for immune thrombocytopenia. Blood Advances, 2019;3(23), pp.3829–3866. Available at: 10.1182/bloodadvances.2019000966 PMC6963252 31794604

[2] Arreola-Diaz R, Majluf-Cruz A, Sánchez-Torres LE, Hernández-Juárez JG. The Pathophysiology of The Antiphospholipid Syndrome: A Perspective From The Blood Coagulation System. Clin Appl Thromb Hemost. 2022;28: 10760296221077944. 10.1177/10760296221088576 PMC8950029 35317658

[3] Tohidi-Esfahani I, Mittal P, Isenberg D, Cohen H, Efthymiou M. Platelets and thrombotic antiphospholipid syndrome. J Clin Med. 2024;13(3):741. 10.3390/jcm13030741 PMC10856779 38337435

[4] Napolitano M, Lucchesi A, Napolitano MS. Immune thrombocytopenia in antiphospholipid syndrome: is it primary or secondary? Biomedicines. 2021;9(9):1170. 10.3390/biomedicines9091170 PMC8472578 34572358

[5] Galarza D, Radin M, Cecchi I, Morotti A, Russo I, Camerlo S, Barinotti A, Fenoglio R, Sciascia S. Thrombocytopenia in antiphospholipid syndrome: predictors, prognostic implications, and thrombotic risk in a large cohort study. Thromb Res. 2026;257: 109544. 10.1016/j.thromres.2025.109544. 41297380

[6] Puavilai T, Thadanipon K, Rattanasiri S, Ingsathit A, McEvoy M, Attia J, Thakkinstian A. Treatment efficacy for adult persistent immune thrombocytopenia: a systematic review and network meta-analysis. Br J Haematol. 2019;186(3):460–72. 10.1111/bjh.16161 PMC7003949 31423574

[7] Knight JS, Kanthi Y. Mechanisms of immunothrombosis and vasculopathy in antiphospholipid syndrome. Semin Immunopathol. 2022;44(3):347-362. 10.1007/s00281-022-00916-w PMC8816310 35122116

[8] Pavord S, Scully M, Hunt B, Lester W, Bagot C, Craven B, Rampotas A, Ambler G, Makris M. Clinical features of vaccine-induced immune thrombocytopenia and thrombosis. N Engl J Med. 2021;385(18):1680–9. 10.1056/NEJMoa2109908 PMC10662971 34379914

[9] Shi Y, Jiang H, Zhao Y, Zhao J, Li M, Zeng X. Immune thrombocytopenia could be an independent clinical phenotype of antiphospholipid syndrome: a prospective cohort study. Rheumatol Ther. 2023;10(3):649-658. 10.1007/s40744-023-00538-0 PMC10140258 36807880

[10] Petito E, Gresele P. Vaccine-induced immune thrombotic thrombocytopenia two years later: should it still be on the scientific agenda? Thromb Haemost. 2023;123(9):1059–62. 10.1055/a-2107-0891. 37285904

[11] Zuily S, Cervera R, Foret T, Bertocchi S, Tincani A. Thrombocytopenia in antiphospholipid syndrome: is anticoagulation and/or antiaggregation always required? Autoimmun Rev. 2023;22(8):103417. 10.1016/j.autrev.2023.103417 37619905

[12] Tiede A, Sachs UJ, Czwalinna A, Werwitzke S, Bikker R, Krauss JK, Donnerstag F, Weißenborn K, Höglinger G, Maasoumy B, Wedemeyer H, Ganser A. Prothrombotic immune thrombocytopenia after COVID-19 vaccination. Blood. 2021;138(4):350-353. 10.1182/blood.2021011958 PMC8084604 34323939

